# Medical News and Miscellany

**Published:** 1896-12

**Authors:** 


### Medical News and Miscellany.

### Information Wanted.

BROOKLYN, N. Y., Nov. 21, 1896.

*To the Editor:*—Who was the writer of the following lines? Can any of our readers cite for an inquiring friend their place of publication:

"God and the doctor we alike adore,
But only when in trouble, not before,

The trouble o'er, both are alike requited;
God is forgotten and the doctor slighted."
—*R. M. W., in Journal A. M. A.*

We pass.

---

**Death of Dr. Letcher.**—The many friends of Dr. J. S. Letcher, of Dallas, will be shocked to learn of his death. He died of intestinal obstruction, December 2nd, inst., at his home in Dallas.

---

**Married.**—At Temple, Tex., November 26th ult., Mr. John Curtis Mitchell to Miss Scott Talley, daughter of Dr. R. P. Talley—all of Temple. The JOURNAL acknowledges the courtesy of cards.

---

**Let us hear** from you, doctor, with a small little remittance. Christmas is coming, and Betty and the baby need shoes and stockings, and "the north wind doth blow, and we shall have snow, and what will poor Robin do" *then*,—unless our subscribers ante up for back raitons.

---

**Dr. W. H. Monday,** of Terrell, Tex., one of the most distinguished physicians of North Texas, in renewing his subscription to the JOURNAL for the twelfth consecutive year, writes: "Let me thank you for, and congratulate you on, publishing the best medical journal in Texas." Thanks, Doctor. *That* is *appreciation* which makes us feel proud.

---

**Personal.**—My Dear Doctor: If you are a real friend of the "Red Back," and want to do us a small favor, and do yourself good at the same time, for it will be to your interest, write —even a postal will do, but preferably a letter—to Kutnow Bros., Nos. 62-64 Lafayette Place, New York, for a sample and literature as per advertisement in this issue for the first time. Do it; we will explain later.

DANIEL & HUDSON.

---

**Dr. C. M. Rosser** has sent in his resignation as Superintendent of the State Lunatic Asylum, at Terrell (North Texas hospital for insane), wonder what's the row this time. We are sure it is not another Simpson case. Dr. F. S. White, of Terrell, who was superintendent of the State lunatic asylum at Austin four years, and assistant physician at the Terrell branch State asylum, will most likely be appointed to the position, as a more experienced or better qualified man for the place can hardly be found.

---

**The Pan-American Medical Congress,** held at Mexico City last month, was fairly attended, there being a respectable representation from Texas. Our junior attended, but we have not been able to induce him to "write up" the meeting. He says "it was all Spanish," and, consequently, those who do not understand the language were "not in it." One cannot help being reminded by these exchange of international courtesies, of the fox and the stork. When the fox dined with the stork, he was regaled with soup served in bottles; he reciprocated the courtesy, and had the stork to dine with him, when he set out his soup in tin plates. We had the Spaniards with us at Washington, and the papers were read in English. At Mexico they were read in Spanish.

Socially, we learn, the occasion was lovely. We, ye senior, didn't go—(as the Irishman said, it is as much as we can do to stay here)—hence, we can not give a description of t[illegible]e occasion.

